# What We Can Learn From Cumulative Numbers of Vesicular Release Events

**DOI:** 10.3389/fncel.2019.00257

**Published:** 2019-06-21

**Authors:** Takafumi Miki

**Affiliations:** Graduate School of Brain Science, Doshisha University, Kyoto, Japan

**Keywords:** synapse, neurotransmitter release, quantal analysis, release site, vesicle recruitment

## Abstract

Following action potential invasion in presynaptic terminals, synaptic vesicles are released in a stochastic manner at release sites (docking sites). Since neurotransmission occurs at frequencies up to 1 kHz, the mechanisms underlying consecutive vesicle releases at a docking site during high frequency bursts is a key factor for understanding the role and strength of the synapse. Particularly new vesicle recruitment at the docking site during neuronal activity is thought to be crucial for short-term plasticity. However current studies have not reached a unified docking site model for central synapses. Here I review newly developed analyses that can provide insight into docking site models. Quantal analysis using counts of vesicular release events provide a wealth of information not only to monitor the number of docking sites, but also to distinguish among docking site models. The stochastic properties of cumulative release number during bursts allow us to estimate the total number of releasable vesicles and to deduce the features of vesicle recruitment at docking sites and the change of release probability during bursts. This analytical method may contribute to a comprehensive understanding of release/replenishment mechanisms at a docking site.

## Introduction

Synaptic vesicles fuse with the presynaptic membrane to release neurotransmitter into the synaptic cleft in a specific structure called active zone (AZ), where each AZ contains one or multiple vesicular docking/release sites (DSs). The DS number corresponds to the maximum number of vesicular release events following an action potential (AP) (review: Pulido and Marty, 2017). The number of released vesicles follows Poisson statistics under conditions of low release probability (e.g., low calcium and high magnesium concentrations extracellularly; Del Castillo and Katz, 1954). However, it was hypothesized that release with normal release probability under physiological conditions is a binomial process (Katz, 1969). Based on this hypothesis, the number of DSs have been estimated by fluctuation analyses using the peak amplitude of synaptic responses (Silver et al., 1998; Clements and Silver, 2000). Assumptions and corrections for heterogeneous release probability and quantal size within and across synapses, synaptic jitter, and quantal size distortion by desensitization and saturation of postsynaptic receptors, are needed to make an accurate estimate of synaptic parameters (DS number *N*, release probability per AZ *P*, and quantal size *Q*; Silver et al., 1998). An alternative approach, instead of using peak amplitudes of synaptic responses from multiple synapses, is to use for variance-mean analysis the number of vesicular release events detected from synaptic responses recorded from synapses having a single presynaptic AZ and a single postsynaptic density (‘simple synapses’: Malagon et al., 2016). In this alternative analysis, the parameter value for *Q* in the analysis using peak amplitude of synaptic response is 1 since the number of vesicular events is used. In this review, I will focus on the newly developed method to count vesicular events at single synapses and I will introduce analyses and model simulations to extract valuable information related to the number of DSs, the vesicle recruitment process, and the pool of releasable/suppliable vesicles. This approach allows us to predict a detailed DS model at a given synapse. Especially the mechanism of vesicle replenishment and the pool size of suppliable vesicles to DS are critical for short-term plasticity and sustained transmission. Experimental manipulations with in-depth electrophysiological examinations have uncovered changes and molecular/morphological correlates of synaptic parameters in DS models at synapses of the mammalian central nervous system (CNS) (e.g., Silver et al., 2003; Lou et al., 2005; Neher and Sakaba, 2008; Hallermann et al., 2010; Südhof, 2012; Liu et al., 2014; Chen et al., 2015; Montesinos et al., 2015; Jackman et al., 2016). More detailed description of DS models by the developed method may more clearly define molecular/morphological correlates of synaptic parameters. In addition, nanometer-resolution observations in mammalian CNS using super-resolution imaging and electron microscopy have revealed synaptic vesicle movement in presynaptic terminals (Midorikawa and Sakaba, 2015, 2017; Rothman et al., 2016; Maschi and Klyachko, 2017; Chang et al., 2018; Kusick et al., 2018), and distribution of synaptic vesicles and AZ proteins (Siksou et al., 2007; Imig et al., 2014; Nakamura et al., 2015; Tang et al., 2016), paving the way for a comparison between data and DS model simulations for a comprehensive understanding of vesicle release/replenishment at DSs if the vesicle recruitment process and the pool size of releasable/suppliable vesicles can be predicted accurately by the new method.

## Counting SV Release

A simple approach for estimating synaptic parameters using fluctuation analysis is to utilize the number of released vesicles at single AZs instead of PSC amplitude, as the former method needs less corrections and assumptions than the latter. Recently we have developed a new method to detect individual vesicular release events using EPSCs recorded in simple synapses (Malagon et al., 2016). One requirement of the method is that PSCs should originate from a single AZ where unitary postsynaptic responses have little variability. Sharp synaptic responses, like those provided by activation of AMPA receptors, are optimal because they provide excellent time estimates of vesicular release events. While these conditions restrict the applicability of the method, the method is advantageous as it can cancel out receptor desensitization and saturation. Additionally, heterogeneity of quantal size and release probability, either within a synapse or across synapses, and asynchrony of vesicular release due to synaptic jitter, do not need to be taken into consideration in a fit of variance-mean plots. Altogether the method allows us to perform quantal analysis in a simple manner by using the number of release events, although some corrections are still required due to the time resolution of event detection (0.2 ms: Malagon et al., 2016). Although deconvolution techniques applied to large synapses provide valuable information, notably concerning mean values of vesicular release event number (Sakaba, 2006), obtaining variance values of the event number require corrections for heterogeneities of postsynaptic responses among AZs in the large synapses. The estimation error for variance in large synapses is further increased by confounders such as postsynaptic receptor desensitization and saturation, and heterogeneities of release probability among AZs. Additionally, the error is accumulated in cumulative number of vesicular events in a train (see next chapter). By contrast, our method essentially takes advantage of the quantal nature of synaptic currents to round up numbers associated with small signals. This eliminates uncertainties linked to quantal size variations, and results in a significant improvement of the accuracy of the results. Hence our method using simple synapses offers a large benefit for quantitative analysis.

The binomial model of vesicular release classically predicts a parabolic variance-mean relationship for synaptic response fluctuations (Katz, 1969). In variance-mean plots of release events numbers, the model parabola has an initial slope of 1, whereas its slope equals the quantal size in the classical analysis using PSC peak amplitudes, and it intersects the *x* axis at the origin and at the DS number, *N*, according to the following formulas:

v⁢a⁢r⁢=⁢N⁢P⁢(1-P)

m⁢e⁢a⁢n⁢=⁢N⁢P

so that:

v⁢a⁢r⁢=⁢m⁢e⁢a⁢n⁢(1-m⁢e⁢a⁢n/N)

where *var* and *mean* are the variance and mean of the number of vesicular release events, *N* is the DS number, and *P* is the release probability per DS. Note that in this formula, the parameter for quantal size *Q* does not appear since event number is used instead of PSC amplitude.

**FIGURE 1:**
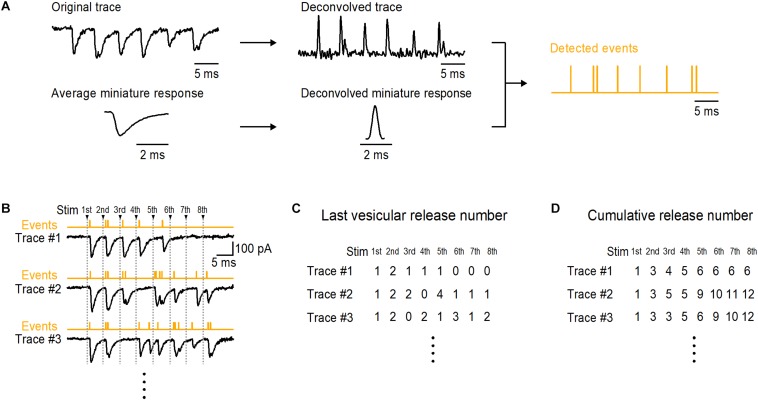
Last and cumulative number of detected vesicular release events in a train at a simple synapse. **(A)** A brief flowchart shows a process to detect vesicular release events by decomposition of postsynaptic currents at a simple synapse. An original trace and miniature responses are obtained in a same experiment. The trace and average miniature response are deconvolved by a multi-exponential curve that is fitted to the average miniature response, producing a deconvolved trace and a deconvolved miniature response (spike). After fitting the deconvolved trace with appropriately time-positioned and vertically scaled spike, a series of events are detected. In detected events, vertical lines show time positions of the individual events. **(B)** Examples of traces in response to 8 APs at 200 Hz at a single PF-MLI synapse. Decomposition of EPSCs provides event detection (see Figures 1, 3 in Malagon et al., 2016). Counted numbers of vesicular events in response to each stimulus in each train are shown in **(C)**; these numbers are called ‘last vesicular release number’ in this report. The cumulative numbers of events for each stimulus are shown in **(D)**.

Malagon et al. (2016) established this method at parallel fiber (PF)-molecular layer interneuron (MLI) synapses in the cerebellum. PFs contact MLIs at single synapses having almost always one AZ (Xu-Friedman et al., 2001). Thus single PF or single granule cell stimulation allows us to obtain responses from a single synapse (Malagon et al., 2016; Miki et al., 2017). When stimulating a PF-MLI synapse with an AP train, delayed release representing desynchronized miniature responses follows synchronized responses (Atluri and Regehr, 1998). By using repetitive trains of stimulations at a PF-MLI synapse, one can obtain from delayed release the average shape of miniature responses as a template of a unitary vesicular release event, as well as synaptic responses during AP trains. For decomposition of recorded EPSCs by the method, obtaining mEPSCs pertaining to the synapse under study is necessary because amplitude and time course of miniature events vary significantly among simple synapses (Auger and Marty, 1997; Crowley et al., 2007). In addition to obtaining miniature responses from asynchronous release such as delayed release, one could obtain miniature responses by lowering release probability that provide one/a few vesicular responses with frequent failures of the responses by stimulation in a given synapse. Direct recording, spot Ca^2+^ uncaging with appropriate flash intensity, and local application of α-Latrotoxin at a single bouton may also be able to provide miniature responses in a simple synapse (Auger and Marty, 1997; Trigo et al., 2012; Kawaguchi and Sakaba, 2017). After averaging mEPSCs, we fitted the average with a triple-exponential curve. Using the triple-exponential curve as a template, we applied deconvolution to the trace and the mEPSC average, thereby producing the deconvolved trace containing a series of spikes and the deconvolved mEPSC being a single ‘spike’ (Figure 1A). Fitting the deconvolved trace with vertically scaled spikes allowed us to detect vesicular release events in the trace. Decomposition of postsynaptic current into the contributions of individual vesicle events by methods including our method is based on the assumption that each vesicular event is a scaled version of a fixed template having the shape of mEPSCs (Clements and Bekkers, 1997; Andor-Ardó et al., 2012; Pernía-Andrade et al., 2012). After converting synaptic responses to vesicular release events by the decomposition of EPSCs, variance-mean analysis of the events reveals 3–10 DSs at one synapse (Malagon et al., 2016), a value comparable to that obtained by EPSC fluctuation analysis (Schmidt et al., 2013; Ishiyama et al., 2014).

## Stochastic Properties of Cumulative Release Number

**FIGURE 2:**
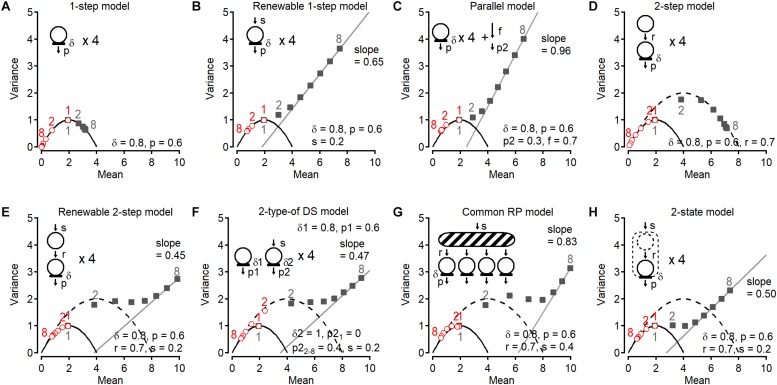
Variance-Mean plots of last release number and cumulative release number in various models. A series of Monte Carlo simulations of variance-mean plots of last release number (red, open circles) and cumulative release number (gray, filled squares) (Miki et al., 2016). Synapses are subjected to trains of 8 APs at 200 Hz. In all panels, a parabola having *N* of 4 is shown. In **(D–H)**, a parabolic curve having *N* of 8 is also shown. In **(B,C,E–H)**, last two or three points for cumulative number were fitted with a line. Corresponding stimulus numbers for the plots of the last release number (red, open symbols) and of the cumulative release number (gray, closed symbols) are shown. **(A)** Simple 1-step model. There are four independent DSs that are occupied by synaptic vesicles with an initial occupancy δ = 0.8 before the 1st stimulus. Docked vesicles have a release probability *p* = 0.6 per DS, for each stimulus. Once docked vesicles are released, no DS replenishment occurs. **(B)** In the renewable 1-step model, a replenishment step is added to the 1-step model in **(A)**. Emptied DSs are replenished from an infinite vesicle pool with a transition probability *s* = 0.2. **(C)** In the parallel model, in addition to the release from four DSs, release occurs as a Poisson process. **(D)** 2-step model. There are four replacement sites paired to four DSs. Once a DS becomes empty, a vesicle is supplied from the paired replacement site with a transition probability *r* = 0.7. There is no recruitment mechanism once a replacement site is depleted. **(E)** In the renewable 2-step model, a replenishment step with rate constant *s* is added to the 2-step model. **(F)** In this model (one-step model + renewable one-step model), there are four 1-step DSs and four renewable 1-step DSs. Parameters in the one-step model are the same as those in **(A)**. **(G)** In another type of DSs, no release occurs upon the 1st stimulus (*p2*_1_ = 0) but release occurs with a probability *p2*_2–8_ = 0.4 from the 2nd stimulus. **(H)** 2-state model. There are two states of vesicle docking at DSs. In contrast to replacement sites, vesicles cannot occupy both states at the same time.

One key benefit of detecting vesicular release events in synaptic responses is to obtain the cumulative number of events in a train. Statistical analysis of the cumulative release number provides valuable information to evaluate release models. In this chapter, I will review several models derived on the basis of a classical binomial model and discuss the stochastic properties of the cumulative release number. Some models are described in a previous paper (Miki et al., 2016). In these models, DSs have a parameter δ that is the probability of occupancy by a synaptic vesicle in the resting state. I call *p* the release probability of a DS that is occupied by a synaptic vesicle. Then the probability of release per DS is δ multiplied by *p* (*P* = δ*p*). A synapse is subject to trains of 8 action potentials (APs) at 200 Hz in all simulations. For each AP number within a train, variance and mean of the last vesicular release number, as well as variance and mean of the cumulative release number are calculated (Figure 1). The last vesicular release number for AP number (**i**) is defined as the number of detected events in response to **i**-th AP in a train (Figures 1B,C). The cumulative release number for AP number (**i**) is the total number of detected events in response to 1st-to-**i**-th APs in a train (**i** > 1; Figure 1D). The first point for cumulative release number (**i** = 1) is always the same as that for last release number (Figures 1C,D). These simulated results are plotted for each model in Figure 2; numbers next to individual points indicate AP numbers in red and gray for the last and cumulative release number, respectively. In all models, a DS number *N* of 4 is assumed except for two-type-of DS model (model (vi) below). Therefore, the plots for the last release number can be fitted with a parabola with *N* = 4 in models (i), (ii), (iv), (v), and (viii).

### (i) One-Step Model

In this very simple model, there are four DSs that cannot be replenished with vesicles once they become empty (Figure 2A). In this case, all variance-mean points are located on a parabola with *N* = 4. The last vesicular release number follows a binomial process as expected. Since no replenishment takes place at DSs, the cumulated release number fluctuates with a maximum value of 4 among trains. Hence cumulative release points are also located on the same parabola (*N* = 4). Since some releasable vesicles are consumed for each stimulus number, while their maximal number remains limited to 4, the plot for the last release number goes toward the origin as stimulus number increases (open symbols). By contrast, cumulative release numbers move toward the *N* value on the *x* axis (closed symbols).

### (ii) Renewable One-Step Model

In this model, an empty DS is replenished with a rate constant *s* (Figure 2B). The number of release events follows a binomial distribution (*N* = 4) as expected, while the cumulative number plot deviates from the parabola starting at the 2nd point, and becomes linear starting at the 4th point. The slope of the regression line (0.65) is less than 1, meaning that release in later stimuli is not a Poisson process.

### (iii) Parallel Model

In contrast to model (ii), in the parallel model release occurs following a Poisson process at DSs or somewhere else in the AZ, in addition to release at DSs without vesicle replenishment (Figure 2C). In this model, the vesicular pool is replenished following a Poisson process with a probability of *f* per interspike interval, and vesicles in the pool release with a probability of *p2*. In this case, the plot for cumulative number deviates from the parabola, as we see in (ii), but the slope is now close to 1, showing that the release later in the stimulation is a random process described by a Poisson distribution. The plot for the last release number is close to the parabola *N* = 4. However, since release takes place in parallel following a Poisson process, a slight deviation from the parabolic curve occurs.

### (iv) Two-Step Model

This model introduces an additional site, called replacement site, for each DS (Figure 2D). Once a DS becomes empty, a vesicle coming from the associated replacement site replenishes the DS with a rate constant *r*. The last release number follows a binomial distribution with *N* = 4, while the variance for the cumulative release number increases at the 2nd point and approaches a second parabola with *N* = 8, corresponding to the sum of the numbers of DSs and replacement sites. Later points follow the second parabola. A fit of 2nd–8th points for cumulative release number with a parabolic curve shows a maximum mean value of 7.89, close to 8. Therefore, the total number of sites can be obtained from variance-mean plots of the cumulative release number, in addition to the number of DSs from those of the last release number.

### (v) Renewable Two-Step Model

This model is similar to the previous model (iv), except that a recruitment step is added to refill the replacement site (Figure 2E). The plot for cumulative release number comes close to the second parabola *N* = 8 at 2nd and 3rd points as in (iv), however, according to this recruitment step it deviates later from the parabola. At the end of the train the plot becomes linear with a slope of 0.45, <1, meaning that release in later stimuli is not a Poisson process as shown in the scheme for the model. Last release numbers follow a binomial process. Because the statistical features of cumulative release were similar for glutamatergic and GABAergic synapses on MLIs (Miki et al., 2016), this renewable two-step model was proposed as a potential generally valid release model. For glutamatergic synapses on MLIs, the two-step model indicated that low δ (= 0.3–0.45) and large *r* (= 0.6) produced initial facilitation followed by depression without change of *p* (= 0.7; Miki et al., 2016). Actin- and myosin-disrupting drugs abolished the initial facilitation, suggesting actin- and myosin-dependent vesicle recruitment to DS. For GABAergic synapses on MLIs, small *r* (= 0.15) produced depression. Interestingly, after prolonged presynaptic depolarization, synaptic responses showed an unusual form of depression/facilitation sequence that could be accounted for by the two-step model with low occupancy of replacement site (ρ = 0.2; Pulido and Marty, 2018). Furthermore, this model can predict a large variety of time-dependent changes of synaptic strength by various combinations of parameter values (Doussau et al., 2017; Pulido and Marty, 2018) and can reproduce the kinetics of release (Miki et al., 2018).

### (vi) Two-Type-of DS Model

A different model also shows similar statistical properties of the cumulative release number. In this model derived from previous reports (e.g., Wu and Borst, 1999; Neher, 2006; Hallermann et al., 2010), two types of independent sites release vesicles with different probabilities (Figure 2F). One is characterized by high release probability without replenishment. The other is replenished from an infinite pool and has a low release probability that is initially 0 and becomes 0.4 starting with the 2nd stimulus. In this case, the cumulative release number plot jumps at the 2nd point to reach the second parabola, and then bends along this parabola. Then the plot gradually departs from the parabola and approaches a linear relation with a slope of 0.47, similar to (v). The last release plot for this model is rather remarkable (see red open circles). Initially second type DSs have a release probability of 0, thus the 1st point of the last release number plot is located on a parabola *N* = 4, whereas the 2nd–8th points are located near a parabola *N* = 8 due to the increase in release probability of second type sites to 0.4. The model is a simplified version compared with the model previously reported. In fact, instead of sudden increase in the release probability from the first to the second stimulus at the second type of sites and no vesicle replenishment to the first type of sites in the simplified model, the reported model assumed a gradual facilitation of the release probability of both type of sites and slow vesicle replenishment from the second to the first sites, reproducing large sets of data (e.g., Pan and Zucker, 2009; Ritzau-Jost et al., 2018). In general, however, parallel models do not provide separate parabolas for last release number and cumulative release number.

### (vii) Common RP Model

In the two-step or the renewable two-step model, a replacement site is associated to each DS. In the present model, a common replacement pool replaces individual replacement sites. Initially four vesicles in the pool are ready to replenish any empty DS (Figure 2G). This pool is replenished up to the permissible number of four with rate constant *s*. As in previous models, the cumulative release number plot jumps from a parabola *N* = 4 to reach a larger parabola representing replacement pool size plus DS number. The slope of the final linear fit is larger than that in the renewable two-step model and in the two-type of DS model. Hence vesicle replenishment through a common replacement pool at DS makes late release resemble a Poisson process. Finally, the last release number plot slightly deviates from the *N* = 4 parabola.

### (viii) Two-State Model

The last attractive model is a two-state model where a DS can accommodate one bound vesicle in two different states (loosely docked state and tightly docked state). This model was proposed based on the evidence that the docked/primed synaptic vesicle state is very dynamic (Neher and Brose, 2018; Figure 2H). In contrast to the two-step model, the two states cannot be simultaneously occupied by vesicles in this model: the second state is replenished from an infinite pool only if no vesicle is present on the site. In this model, the cumulative release number plot does not jump to a larger *N* parabola. Rather, it gradually departs from the parabola with *N* = 4, eventually approaching a linear relation with a slope of 0.50.

The plots for the cumulative number among models (v), (vi), and (vii) are similar in shape. However the slope of the later points in model (vii) is larger than in models (v) and (vi), indicating more random release later in a train. In addition, a discrepancy between model (v) and (vi) appears in the plot for the last release number (red circle #2). Likewise, in the case of another proposed model where new release site recruit between stimulations instead of replenishment of vesicles to vacant DS (Valera et al., 2012; Brachtendorf et al., 2015), the second point for the last release number shift to a bigger parabola than that for the first point. The increase in *N* arises from the fast recruitment of reluctant synaptic vesicles into a fully releasable pool by high-frequency stimulation (Brachtendorf et al., 2015; Doussau et al., 2017).

As described above, there are similarities and discrepancies among models with certain sets of parameter values. In some sets of values the models are hardly distinguished but in the other sets they are distinguishable, since the shape of the plot for the cumulative number changes with the combination of the parameter values. Therefore, models need to be examined in different experimental conditions possibly changing the parameter values.

Stochastic properties of last and cumulative release number at PF-MLI synapses in cerebellum in 2-week-old rats were best fitted with model (v). Further support for this model was provided by pharmacological experiments (Miki et al., 2016). However it remains to be seen whether other synapses may be better depicted with other models of Figure 2.

In summary, when performing variance-mean analysis, the last release number provides information about the number of DSs and the release probability per DS; examination of the variance-mean plot for this number during an AP train provides information on whether release follows a binomial or a Poisson process. Comparing the statistical properties of the cumulative release number among various models, some features concerning replenishment steps are extracted. In certain models, the variance-mean plot displays a jump between the 1st and the 2nd point, implying the existence of an associated site (replacement site) or of an additional vesicle pool replenishing DSs for consecutive release. By contrast, a continuous increase in variance is displayed in other simpler models, like the renewable one-step model and the parallel model (Figures 2B,C). The slope of the fitted line for later points also gives indications concerning the docking/release model. A slope of 1 indicates that late release obeys a Poisson process, while a slope < 1 implies some kinetically limiting steps before DSs. If the cumulative release number plot forms a nearly complete parabola, this indicates little or no replenishment at the DSs. The parabola fit then provides a good estimate of the total number of vesicles associated with the AZ, as exemplified by the one-step model and the two-step model (Figures 2A,D). Even if there is a replenishment step with some rate constant, depending on the rate, the bended part of the cumulative release number plot may provide a rough estimate of the initial vesicle pool size in the model.

**FIGURE 3:**
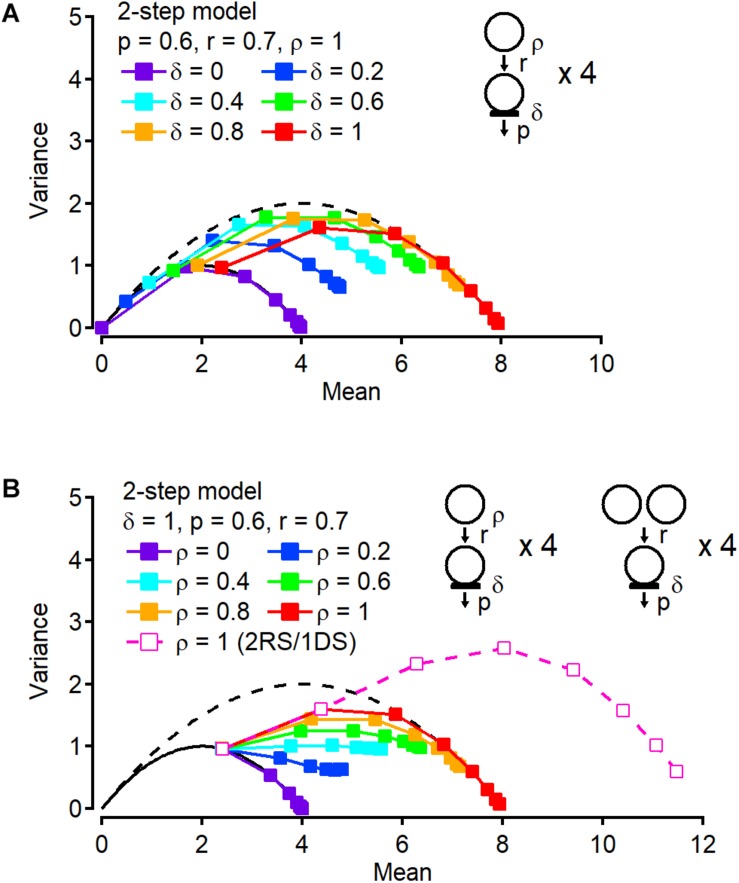
Occupancy at docking and replacement sites affects stochastic properties of cumulative release number. **(A)** Variance-mean plots of cumulative release number with variable occupancy δ at DSs. Occupancy ρ at replacement site is fixed to 1. **(B)** Instead of changing δ, occupancy ρ is changed (filled squares). A variance-mean plot from the model in which there are two replacement sites (RSs) paired to one DS is also shown (open squares).

How reliably can we estimate the total number of releasable/suppliable vesicles using variance-mean plots for cumulative release? Further simulations related to the two-step model are shown in Figure 3 to address this question. In Figure 3A, the occupancy δ of DSs is variable and the other parameters are fixed, including the release probability *p*, the rate constant for the recruitment step *r*, and the occupancy of replacement sites ρ (*p* = 0.6, *r* = 0.7, and ρ = 1). If δ = 0, all points are on the *N* = 4 parabola, corresponding to the exact total number of releasable/suppliable vesicles in DSs and replacement sites. If δ = 1, as shown in Figure 2D, the 1st point is located on the *N* = 4 parabola since release occurs only at four DSs, whereas starting at the 2nd point the plot leaves the *N* = 4 parabola and approaches another parabola with *N* = 8, again corresponding to the total number of releasable/suppliable vesicles. Between δ = 0 and 1, even though the total site number is always 8, the cumulative release plot provides different parabolas with different maximum numbers (*N* = 5.52, 6.72, 7.49, and 7.88 for δ = 0.2, 0.4, 0.6, and 0.8, respectively). Hence not only the site number but also the occupancy of DSs affects the size of the parabola fitting the cumulative release number plot. And the value obtained from the parabola is closer to the total average number of releasable/suppliable vesicles (= *N*δ + *Nρ*) than to the total number of available sites (= 2*N*). Likewise, changes of the occupancy at the replacement site instead of at the DS, affects the cumulative parabola (Figure 3B). In Figure 3B, only *r* is changed and the other parameters are fixed (*p* = 0.6, *r* = 0.7, and δ = 1). If ρ = 0 or 1, the cumulative number plots depict a parabola with *N* = 4 or 8, respectively. If 0 < ρ < 1, extrapolated maximum values from a parabola fit are 5.30, 6.43, 7.25, and 7.75 for ρ = 0.2, 0.4, 0.6, and 0.8, respectively. As above, these numbers are closer to the total average number of releasable/suppliable vesicles (= *N*δ + *Nρ*) than to the total number of available sites (= 2*N*). Finally, doubling the number of replenishment sites/vesicles per DS increases the maximum value of the parabola fit proportionally to the number of additional sites/vesicles (Figure 3B, open symbols). Taken together, a parabolic fit of the cumulative number plot provides us with a rough estimate of the total number of releasable/suppliable vesicles, even though the value that is obtained is a slight over estimation in the cases 0 < δ < 1 and 0 < ρ < 1.

## Concluding Remarks

Recently methods have been developed to count the number of released vesicles at single synapses using deconvolution analysis (Malagon et al., 2016). Reliable detection of vesicular events from synaptic responses allows us to estimate not only the number of DSs as in the conventional fluctuation analysis but also the total number of releasable/suppliable vesicles, and to understand features of the vesicle recruitment/release process by analysis of stochastic properties of cumulative release number. Comparing statistical properties of vesicular event number from data and models allows us to predict a possible DS model for a given synapse. Determining a release model and parameter values for synapses by the methods is crucial for understanding the limiting factor for the synaptic properties, such as short-term plasticity. Applying these analyses to various types of synapses has the potential to provide a comprehensive understanding of release models among synapses. Furthermore, the method of detecting individual vesicular release events, like the previously developed deconvolution analysis (Neher and Sakaba, 2001), also provides timing of the events (Miki et al., 2018). Models can predict speed and time course of vesicle replenishment to DSs, maturation of release machinery on DSs, pool size of synaptic vesicles, and DS number. Those could be investigated by recently developed techniques using super resolution microscopy and electron microscopy. It has been shown that the number of DSs correlates with the number of clusters of AZ proteins such as RIM, Munc13, and Ca^2+^ channels (Tang et al., 2016; Miki et al., 2017; Sakamoto et al., 2018). Thus investigation of these AZ proteins may tell us localization, movement and regulation of DSs. In addition, nano-meter scale movements of synaptic vesicles have been observed after stimulation near the presynaptic membrane, indicating the speed and time course of vesicle recruitment (Midorikawa and Sakaba, 2015, 2017; Kusick et al., 2018). Those studies combined with the model simulation based on the quantal analysis may allow us to see the relationship between parameter values for predicted models and physical observations of DSs and synaptic vesicles. Future studies integrating multiple approaches will provide physical insight into the dynamics of interaction between release machineries and synaptic vesicles at a DS due to consecutive vesicle fusions during neuronal activity and eventually might lead to a unified model among synapses in CNS.

## Data Availability

The raw data supporting the conclusions of this manuscript will be made available by the authors, without undue reservation, to any qualified researcher.

## Author Contributions

The author confirms being the sole contributor of this work and has approved it for publication.

## Conflict of Interest Statement

The author declares that the research was conducted in the absence of any commercial or financial relationships that could be construed as a potential conflict of interest.
